# Absence of erythrocyte sequestration in a case of babesiosis in a splenectomized human patient

**DOI:** 10.1186/1475-2875-5-69

**Published:** 2006-08-04

**Authors:** Ian A Clark, Alison C Budd, Gunther Hsue, Bret R Haymore, Alina J Joyce, Richard Thorner, Peter J Krause

**Affiliations:** 1 School of Biochemistry and Molecular Biology Australian National University, Canberra, ACT 0200, Australia; 2 Departments of Medicine and Pathology, William Beaumont Army Medical Center, El Paso, TX 79920, USA; 3 San Antonio Infectious Diseases Consultants, San Antonio, TX 78229, USA; 4 Department of Pediatrics, University of Connecticut School of Medicine and Connecticut Children's Medical Center, Hartford, CT 06105, USA

## Abstract

**Background:**

The importance of vascular occlusion in the pathogenesis of human haemoprotozoal disease is unresolved.

**Methods:**

Giemsa-stained tissue sections from a human case of *Babesia microti *infection in a splenectomized patient with chronic lymphocytic leukaemia and colon cancer were examined to ascertain the distribution of parasitized erythrocytes within the vascular lumen.

**Results:**

No evidence of sequestration was observed.

**Conclusion:**

This first report on the vascular location of *B. microti *in human tissue suggests that severe multi-organ failure due to babesiosis is independent of sequestration of parasitized erythrocytes. A similar pathogenesis may also cause multi-organ failure in other intraerythrocytic protozoal infections, including falciparum malaria.

## Background

A central issue in the pathogenesis of the disease caused by falciparum malaria is whether vaso-occlusion by sequestered parasitized red cells is the necessary primary event in cerebral malaria and in multi-organ failure. The alternative view is that these changes are set in motion by the toxic effects of excessive release of inflammatory cytokines, with locally enhanced sequestration being a secondary event that sometimes focuses inflammatory mediators and exacerbates local pathology. The topic has recently been extensively reviewed [1,2].

Babesiosis is an emerging haemoprotozoan tick-borne disease often associated with symptoms that are similar to those of falciparum malaria [3,4]. Complications can include altered mental status, adult respiratory distress syndrome, renal insufficiency, disseminated intravascular coagulation (DIC), gastrointestinal bleeding and multi-organ failure [3,4]. The traditional view of these changes, when seen in falciparum malaria, is that they are precipitated by hypoxia secondary to obstructive sequestration [2,5]. Thus, the same constellation of pathologic events in human babesiosis could imply that sequestration also drives the pathophysiology of this disease. There is, however, no information on this crucial question. An answer was, therefore, sought through examining sequestration of *Babesia microti*-infected erythrocyte sequestration from a limited autopsy of a human fatality in which babesiosis was part of a complex diagnosis.

A sixty-seven year old male with chronic lymphocytic leukaemia and colon cancer, who had undergone splenectomy, experienced a *B. microti *infection that was confirmed by thin blood smear identification of *B. microti *DNA by PCR and a four-fold rise in anti-*B. microti *antibody titre. He received two courses of clindamycin and quinine over a two month period with clearing of parasitaemia each time. Four months later he was admitted to hospital with fever, tachypnea and confusion. Confusion continued until coma supervened 19 days later. A diagnosis of babesiosis was made by identification of babesia on blood smears (2 to 3% parasitaemia), amplification of babesia DNA by PCR, and detection of anti-babesia antibody in serum. The patient was treated with clindamycin and quinine, azithromycin and atovaquone and a second round of clindamycin and quinine over the course of three weeks with no improvement in parasitaemia. He developed DIC and became comatose after suffering a cerebellar brain haemorrhage. He expired two days later. Immediately following death, tissue samples from brain (cortex, basal ganglia, pons, leptomeninges, cerebellum and spinal cord), lung, liver and kidney were collected into 10% formalin. Multiple sections from 3–6 paraffin blocks of each sampled tissue were stained with Giemsa and examined extensively by two independent operators to locate parasites. In particular, information was sought to determine whether the parasitized erythrocytes were randomly located within the lumen of blood vessels or showed any tendency to attach to vascular endothelium. Sections were searched over several days until about three hundred parasitized erythrocytes were seen. None were close enough to vascular walls to suggest any degree of sequestration. In terms of sightings per unit time, parasites were about five times more commonly observed in the brain vasculature than elsewhere. The cerebellum haemorrhage site and engorged meningeal vessels provided a much larger number of erythrocytes to peruse, and parasite density within the erythrocyte population in these tissues was equally low as elsewhere. Previously examined sections of *B. microti*-infected tissue of CBA/Ca mice were reviewed, confirming that even at very high parasite densities there was no tendency for parasitized red cells to sequester in this model.

Another haemoprotozoan parasite, *Plasmodium falciparum*, sequesters in *Aotus sp*. [6] as well as in man. Since late *P. falciparum *trophozoites and schizonts in peripheral smears are extremely rare, erythrocytes containing these stages evidently sequester in deeper vessels, even in mild human infections. Hence erythrocyte sequestration following parasite invasion appears to be a characteristic of the parasite rather than the host species, or the degree of host illness. This implies that the non-sequestration observed in this single splenectomised case of human *B. microti *infection will prove to be an equally constant feature of this parasite, as it is in mice, and irrespective of parasite density or its degree of contribution to the fatal outcome. Likewise, should the apparent absence of *B. microti*-infected red cell sequestration in this patient prove to be a general feature of all human babesial infections, a mechanism other than sequestration would be required to explain the multi-organ pathology that may occur during this disease. Although a case of *B. microti *infection in a patient with an intact spleen has yet to be examined histologically for parasite location, this parasite is recorded as causing multi-organ pathology in both intact and splenectomized individuals [7,8]. Thus a difference between sequestration in intact and splenectomized hosts, as occurs in *P. falciparum*-infected *Saimiri sciureus *[9] (and to an extent in human cases [10]) is not required to infer from our case that non-sequestering *B. microti *can cause multi-organ pathology in man.

This first report on the vascular location of *B. microti *in human tissues suggests that severe multi-organ failure due to human infection by this parasite is independent of sequestration of parasitised erythrocytes. As reviewed in reference 1, vital organ sequestration can be vanishingly rare in fatal *P. falciparum *cases, even though adherence in some tissue is an inevitable characteristic of the later half of the erythrocytic cycle of this infection, irrespective of its contribution to death. Indeed, there is a report of two European adult deaths from fulminant untreated falciparum malaria, without discernible sequestration in the vital organs [11], that might plausibly have been due to systemic inflammatory causes. As reviewed for falciparum malaria [1], complications of human *B. microti *infection could, therefore, prove to be mediated by the same inflammatory processes as are accepted for bacterial sepsis and severe influenza [12].

## Authors' contributions

Ian Clark initiated the project, helped plan it, and examined sections. Alison Budd stained the sections and examined them independently. Gunther Hsue managed the patient's anti-babesial regimens during his final hospital course. Bret Haymore assisted in managing the patient in the intensive care unit, performed the medical chart review and provided the clinical details of the case. Alina Joyce performed the autopsy. Richard Thorner made the original diagnosis of babesiosis and provided the first two courses of anti-babesial therapy. Peter J. Krause assisted in the management of the patient and helped plan and carry out the project.
